# Genetic insights into schizophrenia: ERBB4 and GABRB2 polymorphisms in the Lebanese population

**DOI:** 10.1016/j.ibneur.2025.08.024

**Published:** 2025-08-29

**Authors:** Jeanne d’arc Bacha, Nabiha Dannawi, Dana Yassine, Chadia Haddad, Georges Haddad, Dory Hachem

**Affiliations:** a Department of Public Health Genetics, Faculty of Public Health, Jinan University, Tripoli, Lebanon; b Research Department, Psychiatric Hospital of the Cross, Jal Eddib, Lebanon; c Institut National de Santé Publique, d’Épidémiologie Clinique et de Toxicologie-Liban (INSPECT-LB), Beirut, Lebanon; d Inserm U1094, IRD UMR270, EpiMaCT Epidemiology of Chronic Diseases in Tropical Zone, University of Limoges, Limoges, France; e Faculty of Public Health, Lebanese University, Fanar, Lebanon; f School of Medicine and Medical Sciences, Holy Spirit University of Kaslik, P.O box 446, Jounieh, Lebanon; g Faculty of Medicine and Medical Sciences, University of Balamand, Koura, Lebanon

**Keywords:** Schizophrenia, ERBB4, GABRB2, Environmental factors, Genetic, Lebanese population

## Abstract

**Background:**

The ERBB4 and GABRB2 genes have been given their keen roles in various neurological phenomena, in addition to the involvement of various environmental factors.

**Aim:**

To investigate the prevalence of ERBB4 rs839523 C/T and GABRB2 rs1816072 T/C polymorphisms in Lebanese patients with schizophrenia.

**Materials and methods:**

A questionnaire-based case-control study recruiting 100 participants of matched characteristics was conducted. A Tetra Primer Amplification Refractory Mutation System Polymerase Reaction (T-ARMS PCR) was performed to evaluate the prevalence of the studied polymorphisms.

**Results:**

There were no correlations found between schizophrenia and the ERBB4 rs839523C/T polymorphism (p = 0.096 for genotypes and p = 0.113 for alleles) or the GABRB2 rs1816072 T/C polymorphism (p = 1.00 for genotypes and p = 0.15 for alleles) in the Lebanese population. However, the logistic regression model showed that the mutant homozygous (TT) genotype of the SNP rs839523 in ERBB4 tended to be significant (p = 0.063) with the schizophrenia status. When considering alleles individually, the logistic regression did not reveal any significant associations; however, the C allele of ERBB4 rs839523 showed a trend toward significance.

**Conclusion:**

Our findings revealed that the mutant homozygous (TT) genotype of the SNP rs839523 in ERBB4 showed a trend toward significance. These findings suggest a potential role for ERBB4 genetic variation in schizophrenia susceptibility, warranting further investigation in larger cohorts.

## Introduction

Schizophrenia is a chronic and severe mental disorder that disrupts an individual’s thoughts, emotions, and behavior. It is characterized by a combination of positive symptoms (such as hallucinations and delusions), negative symptoms (such as social withdrawal and reduced emotional expression), and cognitive impairments. In Lebanon, it is estimated that approximately 50,000 individuals, about 1 % of the population, are affected by psychotic disorders (El-Khoury et al., 2018). While this figure encompasses a broader range of conditions, including schizophrenia, the focus of the present study is specifically on schizophrenia. Nowadays, the disorder typically emerges in late adolescence, and is recognized as a multifactorial disorder resulting from the complex interaction of genetic and environmental factors (Wahbeh and Avramopoulos, 2021). It is now well established that many mental illnesses, including schizophrenia, have a strong genetic component. Among the genes implicated in schizophrenia are gamma-aminobutyric acid A receptor beta 2 (*GABRB2)* and Erb-B2 Receptor Tyrosine Kinase 4 (*ERBB4)*, which encode the Gamma-Aminobutyric Acid Type A Receptor Beta2 Subunit and the Erb-B2 Receptor Tyrosine Kinase 4, respectively. The *GABRB2* gene has been associated with social cognition and altruistic behavior (Tsang et al., 2013), while *ERBB4* plays a key role in neurogenesis and synaptic formation (ALEITAN et al., 2017). Both *GABRB2* and *ERBB4* play pivotal roles in regulating key neurotransmitter systems, namely Gamma-Aminobutyric Acid (GABA), glutamate, dopamine, and neuregulin-1 (NRG1) signaling, all of which are central to the neurobiological mechanisms underlying schizophrenia (Tsang et al., 2013, Banerjee et al., 2010).

The *GABRB2* gene, located on chromosome 5q34, spans approximately 260 kb and comprises 11 exons and 10 introns. It encodes the β2 subunit of the GABAA_AA receptor, which assembles with α and γ subunits to form a 250 kDa receptor belonging to the Cys-loop superfamily (Barki and Xue, 2022, Choi et al., 2022). This receptor mediates GABAergic transmission, the primary inhibitory signaling pathway in the brain, essential for synaptogenesis, neurogenesis, neuronal plasticity, and learning (Barki and Xue, 2022). Several intronic SNPs in *GABRB2*, particularly in the non-coding regions targeting exon 10 (a key site for alternative splicing), have been strongly associated with schizophrenia (Zhao et al., 2009). The SNP rs1816072, identified in German, Japanese, Chinese, and more recently Iranian populations, involves a T/C substitution in intron 8 and may disrupt exonic splicing enhancers or silencers (Liu et al., 2005, Lo et al., 2007a). Functional studies conducted in U.S. populations revealed that individuals with the *GABRB2* C/C (homozygous) and T/C (heterozygous) genotypes exhibited a significant reduction in β2 subunit mRNA levels (Zhao et al., 2007). Several studies have further suggested that specific allelic variants in *GABRB2* may influence schizophrenia-related features, including disease occurrence and age at onset (Lo et al., 2007a, Chen et al., 2009), symptom severity and required antipsychotic dosage, as well as aspects of social cognition (Tsang et al., 2013), although these associations remain inconsistent across different populations.

Similarly, the *ERBB4* gene, located on chromosome 2q34, spans approximately 1.16 Mb and includes 28 exons (ALEITAN et al., 2017). It encodes a 1308-amino-acid receptor tyrosine kinase expressed in dopaminergic neurons, glial cells, and various interneuron subtypes (Mei and Nave, 2014, Iwakura and Nawa, 2013). Upon binding with ligands such as NRG1, ERBB4 activates multiple signaling pathways involved in neurogenesis, synaptic formation, neuronal plasticity, maturation, and migration (ALEITAN et al., 2017). The rs839523C/T SNP in intron 2 has been associated with increased expression of the CYT-1 isoform and a heightened risk of schizophrenia, particularly among Ashkenazi Jewish and Jordanian populations (ALEITAN et al., 2017). This upregulation of the CYT-1 isoform has been linked to altered neuregulin-ERBB4 signaling pathways, which play a critical role in interneuron development and synaptic plasticity. Functional studies suggest that rs839523 may influence alternative splicing of *ERBB4*, providing a mechanistic explanation for its involvement in schizophrenia pathophysiology (Silberberg et al., 2006). *ERBB4* signaling is further implicated in the development and synaptic integration of cortical inhibitory interneurons by regulating the connectivity and function of GABAergic neurons, particularly parvalbumin-positive (PV⁺) interneurons (Wen et al., 2010). This pathway, mediated through neuregulin-1 binding, contributes to maintaining the excitatory/inhibitory balance in the cortex, suggesting functional overlap with GABRB2-mediated GABAergic neurotransmission (Fazzari et al., 2010, Neddens et al., 2011).

Alternatively, it is estimated that 15–40 % of schizophrenia risk can be attributed to environmental factors, many of which remain poorly understood (Robinson and Bergen, 2021). The presence of a family history and being born in the spring are factors beyond individual control that have been proposed as potential contributors to schizophrenia risk, although the seasonal birth effect remains a topic of ongoing investigation and debate (Kendler and Klee, 2024, Wang and Zhang, 2017). Additional associations have been observed with modifiable factors, including smoking, low socioeconomic status, and limited education (Tesli et al., 2022, Ding and Hu, 2021).

While the genetic basis of schizophrenia is still not fully elucidated, growing evidence suggests that multiple rare structural variants may contribute to its onset and progression. Polymorphisms in genes such as *GABRB2* and *ERBB4* have been studied in relation to schizophrenia, yet findings remain inconsistent across populations (Marsman et al., 2014, Hahn et al., 2006, Boulenouar et al., 2022). It is likely that genetic and environmental factors interact in complex ways to influence disease susceptibility. Therefore, the present study aims to investigate the potential association between the *ERBB4* rs839523C/T and *GABRB2* rs1816072 T/C polymorphisms, alongside selected environmental factors, in contributing to schizophrenia risk within the Lebanese population.

## Materials and methods

### Study design

An observational epidemiological case-control genetic study was conducted on 50 patients and 50 controls at the Psychiatric Hospital of the Cross (HPC) in Lebanon. The patients were diagnosed with schizophrenia according to the DSM-V criteria and with a hospitalization period of one year at baseline. The main criterion for inclusion in the study was the occurrence of schizophrenia without a previous head injury. Moreover, they ought to be free from any chronic or neurological disorders besides schizophrenia and with no history of traumatic brain injury. 50 participants for the control group with no neurological, psychiatric, or other chronic disorders, were provided by the Al Hamidi Center. Pregnancy, and breastfeeding, were excluded from this study with consent being a major factor. The cases and control groups were matched by age and gender distribution.

### Ethical statement

The study was approved by the local Ethics Committee of Jinan University's ethical review board (reference number: ERB-FPH-202302) and the Ethics and Research Committee of the Psychiatric Hospital of the Cross (reference number: HPC 001–09–23) in compliance with the Hospital’s Regulatory Research Protocol. An informed consent was obtained from each participant at the beginning of the survey and before having access to the questionnaire and participation. The research involved the use of completely anonymous surveys.

### Sample size calculation

The sample size was calculated using Epi Info software for an unmatched case-control design. Based on a previous study evaluating the relationship between GABRB2 gene polymorphisms and schizophrenia, the rate of the GABRB2 rs1816072 T/T genotype in healthy controls was 42.5 % (Heidari Nia et al., 2022), an expected odds ratio of 3.08, a power of 80 %, and a 90 % confidence level. Under these parameters, the minimum required sample size was 96. In our sample, we have recruited 100 participants to ensure sufficient statistical power. This approach allowed us to adequately power the study while considering the genetic distribution within the target population.

### Demographic information and environmental factors

A questionnaire was used in Arabic, the native language in Lebanon to collect the demographic information and the environmental factors. The questionnaire assessed the demographic data of participants, including age, and gender, and the environmental factors such as tobacco smoking, and physical exercises in addition to the family history of schizophrenia episodes. The data is collected from April until July 2024.

### DNA sampling and genotyping

Blood samples were drawn from 50 patients and 50 healthy, and they were used for DNA extraction and purification to genotype *ERBB4* rs839523C/T and *GABRB2* rs1816072 T/C polymorphisms by T-ARMS-PCR.

### DNA extraction

Genomic DNA was extracted, using Quick-DNA™ Miniprep Plus Kit (Zymo Research, D4068). The Kit is the easiest method for high-yield total DNA extraction that relies on using Zymo-Spin™ IIC-XLR Column in a Collection Tube. 200 µl of blood sample were used for DNA extraction with the addition of 200 µl of Biofluid red followed by 20 µl of Proteinase K. After mixing by vortex thoroughly for 10–15 s and incubating in a water bath at 55˚C for 10 min, 1 vol of Genomic Binding Buffer (420 µl) was added to the digested sample. The mixture was then transferred to a Zymo-Spin™ IIC-XLR Column in a Collection Tube and centrifuged at ≥ 12,000 x g. Several washing steps for the mixture were performed using DNA Pre-Wash Buffer and g-DNA Wash Buffer. Lastly, ≥ 50 µl DNA Elution Buffer was added to the matrix to elute the genomic DNA through incubating for 5 min at room temperature. After centrifugation at maximum speed, the eluted DNA can be used immediately for molecular-based applications or stored at ≤ -20°C for future use.

### Genotyping

The selected polymorphisms were detected for each extracted DNA using the tetra-primer amplification refractory mutation system-polymerase chain reaction (T-ARMS-PCR). This technique uses four refractory primers (inner and outer primers) in a single PCR mixture (Table 1). The 3′ end of the inner primers (inner primer 1 and inner primer 2) were designed so that one set of primers can amplify the normal allele and another can amplify the mutant allele. The single mismatch base is introduced at the 3′ end of the primer (Table 1). This mismatch allows the primer to amplify only a single allele and refractory to the other allele. Although the PCR products were not sequenced, all primers used in this study were carefully designed and evaluated using NCBI Primer-BLAST Software to ensure high specificity to the target regions *(*https://www.ncbi.nlm.nih.gov/tools/primer-blast/*).* The detailed Primer-BLAST outcomes for all primers are provided in Supplementary File 1. The PCR was performed in a 20 µl reaction comprising: 10 µl of Red Taq DNA polymerase (REDTaq® ReadyMix™ PCR Reaction Mix (Sigma-Aldrich, R2523–100RXN), 4 µl of tetra primers (1 µl (0.5 µM) of each primer) (Supplementary file 2 Table S1), 4 µl of PCR water and 2 µl of genomic DNA.

**Table 1:** The primers of the studied polymorphisms for ERBB4 and GABRB2 genes along their products' respective sizes.

Gene	Polymorphisms	Primers	Sequence 5’ to 3’	Product size
**ERBB4**	rs83952**3C**	Forward (inner 1)	TGGCATTTGGATCACATATTACCA**C**	572 bp
		Reverse (outer 1)	ACCAACTTATGGGTGGCCTT	
	rs839523 **T**	Forward (outer 2)	CCTGGCCATCCAGGTTGTTA	951 bp
		Reverse (inner 2)	ACTTATTATTTGCAGATTATTTGCAGCTT**A**	
**GABRB2**	rs1816072 **T**	Forward (inner 1)	GATTCTCATTCCAATGGCAACTCTA**T**	956 bp
		Reverse (outer 1)	GGCCATGCAGAGAGCCTAAT	
	rs1816072 **C**	Forward (outer 2)	ACCATAGACCTCCCCTGTGT	783 bp
		Reverse (inner 2)	TCAAGATCACACAGATGGAAAGTT**G**	

### Electrophoresis

**Fig. 1:**
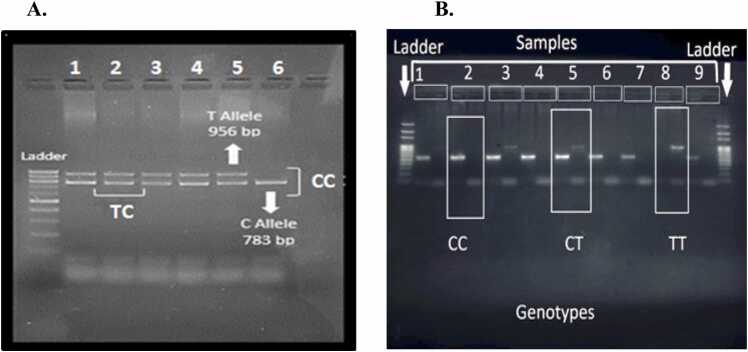
Gel electrophoresis for DNA samples after PCR. (A) All the samples for *GABRB2* from 1 to 5 are heterozygote (TC), except for the homozygous mutant 6 (CC). (B) All genotypes for *ERBB4* are present, 2 is homozygote wild type (CC), 5 is heterozygote (TC), and 8 is homozygote mutant (TT).

Electrophoresis was carried out using the Bio-Rad gel caster at 1.5 % agarose gel (Sigma-Aldrich, A9539–250G) as concentration, which is prepared in 100 ml of 1x TAE (Tris-acetate-EDTA) buffer. 2 µl of ethidium bromide was added to the thawed gel. After the gel is cooled, the DNA ladder (D3687–1VL - Sigma®) is loaded in the first well, and 12 µl of samples (PCR products) in the next wells. All the primers for *GABRB2* (Fig. 1A) were placed in the same tubes, but primers for C and T alleles for *ERBB4* (Fig. 1B) were separated in two different PCR tubes for each individual with the difficulty of reading the heterozygous state on the gel. The bands for each allele were visualized separately under UV light using the ENDURO™ Labnet (UV) Transilluminator for healthy and patient groups.

### Data and statistical analysis

The statistical analysis was carried out using IBM SPSS software, version 25 to verify the involvement of *ERBB4* rs839523C/T and *GABRB2* rs1816072 T/C polymorphisms in the pathophysiology of schizophrenia. In addition, the impact of environmental factors was investigated. The chi-square and Fisher exact tests were used to investigate the association between the disease and the studied polymorphisms and environmental factors. A logistic regression model was conducted, taking the participant status as the dependent variable and the studied polymorphisms as the independent variables adjusted over age and gender. P-values were calculated, and the results were significant when the p-value < 0.05.

## Results

The prevalence of the SNP rs839523 in *ERBB4* was assessed in this study sample (n = 100). The wild-type (CC), heterozygous (CT), and mutant homozygous (TT) genotypes were observed at frequencies of 53 %, 39 %, and 8 %, respectively (Table 2). The allele distribution in the overall population was 72.5 % for the wild-type C allele and 27.5 % for the mutated T allele.

**Table 2:** Distribution Of The Genotype And Allele Frequencies of ERBB4 and GABRB2 Genes Polymorphism Among the Participants of the Study Population.

	**Total**	**Patients with Schizophrenia**	**Healthy Control**	**p-value**
**ERBB4 rs839523**				
Genotypes				
CC	53 (53.0 %)	24 (48 %)	29 (58 %)	0.096
CT	39 (39.0 %)	19 (38 %)	20 (40 %)	
TT	8 (8.0 %)	7 (14.0 %)	1 (2 %)	
Total Number of Participants	100	50	50	
Alleles				
C	145 (72.50 %)	67 (22.0 %)	78 (78.0 %)	0.113
T	55 (27.50 %)	33 (33.0 %)	22 (67.0 %)	
Total Allele Frequency	200	100	100	
**GABRB2 rs1816072**				
Genotypes				
CC	1 (1 %)	1. (2 %)	0 (0 %)	1.000
CT	99 (99 %)	49 (98 %)	50 (100 %)	
TT	0	0 (0 %)	0 (0 %)	
Total Number of Participants	100	50	50	
Alleles				
C	101 (50.5 %)	51 (51.0 %)	50 (50.0 %)	0.150
T	99 (49.5 %)	49 (49.0 %)	50 (50.0 %)	
Total Allele Frequency	200	100	100	

In the schizophrenia group, genotype distribution was as follows: 48 % had the CC genotype, 38 % had CT, and 7 % had the homozygous mutant TT genotype. In contrast, the healthy control group showed 58 % CC, 40 % CT, and 2 % TT. Despite these differences, no significant association was detected between the *ERBB4* rs839523 polymorphism and the disorder in this study population (p = 0.096).

Regarding allele frequencies, the wild-type C allele was more prevalent in the healthy group (78 %) compared to the mutated T allele (22 %). Conversely, in the patient group, the mutated T allele frequency (33 %) exceeded that of the wild-type C allele (22 %). However, this difference did not reach statistical significance (Fisher’s Exact test, p = 0.113).

For the GABRB2 rs1816072 SNP, among the 100 participants, the heterozygous TC genotype was predominant, observed in 99 % of individuals, while only 1 % had the mutant CC genotype. The wild-type homozygous TT genotype was absent. The frequency of the wild-type T allele was 49.5 %, slightly lower than the mutant C allele frequency of 50.5 %.

All healthy participants are heterozygous (100 %) having TC as a genotype. However, the homozygous mutant CC genotype was found only in the patients (2 %), while the homozygous wild-type homozygous TT genotype was found in neither. No significant association was found between rs1816072 in GABRB2 and schizophrenia (p = 1). Allele frequencies in the patient group were 51 % for the C allele and 49 % for the T allele, while in the healthy group, the frequencies were equal at 50 % each (Table 2). These differences were not statistically significant (p = 0.15).

### Demographic characteristics and their relationship to participant status

Based on our analysis, 50 % of the females were diagnosed with schizophrenia compared to 60 % of females in the healthy control group. No significant sex difference was observed between healthy individuals and schizophrenia patients (p = 0.315). Moreover, 65.6 % of schizophrenia patients reported a familial history of psychotic disorders compared to only 2 % in the healthy control group. This study revealed that having a family history with a psychotic disorder is strongly associated with the risk of developing schizophrenia (p < 0.001).

Regarding smoking habits, 62.9 % of patients smoked regularly, while 50 % of healthy controls were smokers. Although the difference was not statistically significant, smoking remains an important risk factor given its related health implications (p = 0.241). In terms of physical activity, the majority of patients (75 %) reported engaging in exercise compared to 44.9 % of healthy controls, approaching statistical significance (p = 0.062).

Finally, the mean age of patients (56.76 ± 11.85 years) and healthy controls (59.26 ± 8.92 years) did not differ significantly (p = 0.236) (Table 3).

**Table 3:** Bivariate analysis taking the participants' status as the dependent variable.

Characteristics	Variables	Total (n)	Healthy n(%)	Patients n(%)	p-value
**Gender**	Male	45	20(44.4 %)	25(55.6 %)	0.315
	Female	55	30(54.5 %)	25(45.5 %)	
**Family History**	Yes	22	1(4.5 %)	21(95.5 %)	< 0.001
	No	60	49(81.7 %)	11(18.3 %)	
**Smoking status**	Yes	47	25 (53.2 %)	22 (46.8 %)	0.241
	No	38	25 (65.8 %)	13 (34.2 %)	
**Exercise**	Yes	31	22 (71.0 %)	9 (29.0 %)	0.062
	No	30	27 (90.0 %)	3 (10.0 %)	
			**Mean ± SD**	**Mean ± SD**	
**Age**		58.01 ± 10.51	59.26 ± 8.92	56.76 ± 11.85	0.236

### Multivariable analysis

A logistic regression model, taking the status of the participants (schizophrenia *vs.* healthy control) as the dependent variable adjusted over age and gender, showed that the mutant homozygous (TT) genotype of the SNP rs839523 in ERBB4 gene tended to significance. The GABRB2 rs1816072 gene did not show any significant effect with the status of the participants (p = 1.000) (Table 4).

**Table 4:** Logistic regression taking the status of the participants (schizophrenia vs healthy control) as the dependent variable.

**Factor**	**OR**	**95 % CI**	**P value**
**ERBB4 rs839523**			
CC	Ref		
CT	0.848	0.366, 1.965	0.701
TT	0.126	0.014; 1.117	0.063
**GABRB2 rs1816072**	0.001	0.0001; 1.000	1.000
**Gender**	1.489	0.649; 3.416	0.347
**Age**	1.019	0.979; 1.060	0.366

Another logistic regression model was conducted with allele variants as independent variables. The results showed that none of the tested alleles reached statistical significance. For ERBB4 rs839523, the C allele was associated with a higher odds of schizophrenia (OR = 6.724; 95 % CI: 0.743–60.862; p = 0.090), although this did not meet the significance level. The T alleles of both ERBB4 rs839523 and GABRB2 rs1816072 showed no significant association with schizophrenia (Supplementary file 2 table S2).

## Discussion

In this study, we examined the involvement of *ERBB4* rs893523C>T SNP in Lebanese schizophrenic patients and found no significant genetic association (p = 0.096). This result contradicts the strong association found in Ashkenazi Jews (p = 0.00024) (Silberberg et al., 2006)*.* Similarly, a conflicting study accomplished in the Jordanian population has proven a significant association of the studied SNP with schizophrenia (p = 0.006) (ALEITAN et al., 2017)*.* The differences in ethnicity and sample size can explain this contradiction. The genotypes of 50 patients and 50 controls were examined in our study; compared to the 380 individuals in the Jordanian sample, 185 of them were cases and 195 were healthy controls which might explain the difference in the results found between the studies (ALEITAN et al., 2017)*.* However, it is elemental to highlight that the Jordanian sample contained only male participants, mainly due to stigma. In contrast, in our work, both genders were recruited equally in patients (25 males, 25 females) and a very close count in matched healthy participants (20 males and 30 females). Nevertheless, consistent with this study's results, no association between the *ERBB4* rs839523C>T and schizophrenia was found in several population samples, such as *Turkish* (Sözen and Kartalcı, 2015), Korean (Bae et al., 2012)*,* Han Chinese (Lu et al., 2010)*,* Bulgarian (Betcheva et al., 2009)*,* Japanese (Shiota et al., 2008)*,* and the United Kingdom (Benzel et al., 2007).

The analysis of the percentage of the three genotypes in the case group in this study is close to the Jordanian study (ALEITAN et al., 2017)*.* This is interesting when taking into consideration the sample size and the demographic differences between the two studies, especially since the studied rs839523 SNP is associated with schizophrenia in the Jordanian study, unlike in this Lebanese one. Surprisingly, by a logistic regression model, we detected that the mutant homozygous (TT) genotype of the SNP rs839523 in ERBB4 gene tended to significance (p = 0.063). Despite not reaching statistical significance, the observed trend is consistent with other research that links ERBB4 to neurodevelopmental processes related to schizophrenia (Benzel et al., 2007, Nicodemus et al., 2010, Nicodemus et al., 2006). In order to verify this possible genetic relationship and investigate the underlying biological processes, more research with larger sample sizes and functional validation is required.

The allele frequencies in this Lebanese population samples recorded similar results found in a European subpopulation (British, England, and Scotland) (database rs839523) (STUDIES, 2010). Interestingly, in our study, the frequency of the T allele in percent (33 %) is higher than the C allele (22 %) in the patients’ group though lacking significance (p = 0.113) contradicts the findings in the control group where the C allele has the higher percentage (78 %) when compared to the T allele (67 %) within the same group. This hints at a possible role for the mutated T allele in schizophrenia, and further investigation is required with a much larger sample size within the Lebanese population.

As for *GABRB2*, it has been implicated with schizophrenia in previous studies. To better understand its role, we must know that genes can affect schizophrenia in three ways and are thus divided into three categories: susceptibility genes, which directly influence the risk of schizophrenia, modifier genes, which influence the severity and appearance of symptoms, and mixed genes, which play a role in both previous mechanisms (Fanous and Kendler, 2008)*.* Our investigated gene, *GABRB2*, and its rs1816072 T > C SNP have been found to impact major neurotransmitter transmission in schizophrenia, promoting it to be considered a susceptibility gene in several populations (Tsang et al., 2013, Heidari Nia et al., 2022, Lo et al., 2007b).

However, among our participants, no significant genotype differences were viewed between the 50 healthy and the 50 patients (p = 1). The results contradict the proposition that *GABRB2* is a susceptibility gene for schizophrenia, at least in our studied population. However, we cannot rule out the importance of the studied polymorphism of the GABRB2 gene among schizophrenic patients in Lebanon due to the limited number of samples. Yet, our results cooperate with another study on Han Chinese, which also rejects *GABRB2* as a susceptibility gene for schizophrenia (Zhang et al., 2018)*.*

Despite *GABRB2* not being a susceptibility gene in the Lebanese population, in Han Chinese and the US populations, *GABRB2* was significantly correlated with both psychosis and altruism, specifically in case of severe psychosis rather than mild psychosis. Add to this, rs1816072 T > C SNP was associated with a high dosage of antipsychotics in US schizophrenic patients (Tsang et al., 2013). Hence, this proves the crucial role of this polymorphism in the health of the central nervous system, especially in the brain. Moreover, a study achieved previously by the same researchers on US genomic samples taken from the same research foundation shows that the rs1816072 T > C SNP was associated with a reduction in the β subunits mRNA (Zhao et al., 2007). This suggests that *GABRB2* could also have a role as a modifier gene, making it a mixed gene.

Several environmental factors related to schizophrenia were evaluated in this Lebanese study population. A significant association was found between having a family history of psychotic disorders and schizophrenia (p < 0.001), 65.6 % of the schizophrenia patients in the sample presented a family history of schizophrenia among other psychotic disorders, this is consistent with the study by Lu Y and colleagues, where family history increases the risk by 10-fold (Lu et al., 2018).

The majority of patients with schizophrenia were smokers however no significant difference was found between smoking and patient participants. These findings are partially consistent with previous reports suggesting a potential two-fold increased risk of developing schizophrenia among smokers (Scott et al., 2018), as well as an association between smoking and the worsening of positive symptoms. In summary, while our study did not find significant associations between the ERBB4 rs839523C/T and GABRB2 rs1816072 T/C polymorphisms and schizophrenia in the Lebanese population, it highlights the substantial role of environmental factors in the disorder's development. Family history and smoking habits are significant contributors to schizophrenia risk, underscoring the complex interplay between genetic predisposition and environmental influences. These findings emphasize the need for larger, more comprehensive studies to further investigate gene-environment interactions in schizophrenia within diverse populations.

Overall, this study contributes to the growing body of research examining the genetic and environmental underpinnings of schizophrenia, highlighting the complex interplay between specific gene variants and potential risk factors. Although the current analysis was limited to two polymorphisms, future studies should consider adopting broader genetic approaches, such as multi-gene analyses, polygenic risk scores (PRS), or genome-wide association studies (GWAS), to better capture the polygenic nature of schizophrenia. Incorporating such models could improve risk prediction and help identify biologically meaningful subgroups of patients. Moreover, expanding the genetic scope, increasing sample size, and integrating detailed environmental and clinical data, including sub-phenotypic information such as age of onset, symptom severity, and treatment response, will be essential to advancing our understanding of schizophrenia’s multifactorial etiology.

## Limitations

Despite the valuable insights provided by this study, several limitations should be acknowledged. First, the relatively small sample size (n = 100) may have limited the statistical power to detect subtle genetic associations, potentially overlooking minor effects of the studied polymorphisms. Second, the case-control design is subject to recall bias, particularly concerning self-reported environmental factors such as smoking habits and family history. Additionally, the study focused on only two genetic polymorphisms, which may not fully capture the complex genetic architecture of schizophrenia. The availability of blood samples in our study offers an important opportunity to broaden future genetic investigations. Expanding the analysis to include additional schizophrenia-associated genes such as DISC1, NRG1, COMT, and DTNBP1 could provide a more comprehensive view of the genetic risk factors relevant to our population. Also, the PCR products were not sequenced, which may limit the verification of the amplified target sequences. The lack of detailed stratification based on schizophrenia subtypes could have masked potential associations. Clinical data such as age of onset, symptom severity, and treatment response were not available in the current study, which limits the ability to explore associations between SNPs and these sub-phenotypes. Finally, environmental factors were limited to a few variables, excluding other relevant influences such as stress exposure, water source, childhood adversity, or substance abuse, which could contribute to disease susceptibility. Future studies with larger, more diverse cohorts and comprehensive genetic and environmental assessments are recommended to validate and expand upon these findings.

## Conclusion

In conclusion, this study explored the association of the *ERBB4* rs839523C/T and *GABRB2* rs1816072 T/C polymorphisms with schizophrenia risk in the Lebanese population. The wild-type TT genotype of *ERBB4* rs839523 showed a trend toward significance, suggesting a potential role in schizophrenia susceptibility. In contrast, the *GABRB2* rs1816072 T/C polymorphism was not associated with the risk of developing schizophrenia. However, our findings highlight the significant influence of family history on schizophrenia susceptibility. The absence of associations with age, gender, smoking, and physical activity further emphasizes the complex, multifactorial nature of the disorder. These results underscore the importance of genetics and environmental risk factors in schizophrenia research and suggest the need for larger-scale studies to explore potential gene-environment interactions in diverse populations.

## Abbreviations

NMDAR, N-methyl-D-aspartate receptor; GABA, Gamma-aminobutyric acid; ERBB4, Erb-B2 Receptor tyrosine kinase 4; GABRB2, Gamma-aminobutyric acid A receptor beta 2; SNP, Single nucleotide polymorphisms; HPC, Psychiatric Hospital of the Cross; PCR, Polymerase chain reaction

## Author contributions

JDB designed the study; JDB, ND, DY drafted the manuscript; JDB, ND, DY, CH carried out the analysis and interpreted the results; DH, CH, GH assisted in drafting and reviewing the manuscript; JDB supervised the course of the article. All authors reviewed and approved the final version of the manuscript.

## CRediT authorship contribution statement

**Jeanne d’arc Bacha:** Writing – original draft, Validation, Supervision, Methodology, Investigation, Conceptualization. **Dana Yassine:** Writing – original draft, Project administration, Methodology. **Nabiha Dannawi:** Writing – original draft, Project administration, Methodology. **Georges Haddad:** Writing – review & editing, Validation. **Chadia Haddad:** Writing – review & editing, Validation, Investigation, Formal analysis. **Dory Hachem:** Writing – review & editing, Supervision.

## Consent for publication

Not applicable.

## Funding

The genetic analysis work was supported by the 10.13039/501100004024Jinan University. No grant ID is applicable for this work

## Conflicts of Interest

The authors have no competing interests to declare that are relevant to the content of this article.

## Data Availability

The datasets generated during and/or analyzed during the current study are available from the corresponding author on reasonable request

## Footnotes

Appendix A Supplementary data associated with this article can be found in the online version at doi:10.1016/j.ibneur.2025.08.024.
