# Supplementary material for: Genetic insights into schizophrenia: ERBB4 and GABRB2 polymorphisms in the Lebanese population
Source: IBRO Neurosci Rep. 2025 Aug 29;19:597–603. doi: 10.1016/j.ibneur.2025.08.024 (PMC12444150; doi:10.1016/j.ibneur.2025.08.024)
Supplement: Supplementary file 1 — Supplementary material [file mmc1.docx]

**Supplementary file 1: Primer-BLAST outcomes**

**rs839523C primer pairs**

Input PCR template: lcl|Query_1

Range: 1 - 9000

Specificity of primers: Primer pairs are specific to input template as no other targets were found in selected database: NCBI Messenger RNA Reference Sequences (Organism limited to Homo sapiens)

Primer pair 1

Forward primer Sequence (5'->3'): TGGCATTTGGATCACATATTACCAC

Forward primer Template strand: Plus

Forward primer Length: 25

Forward primer Start: 926

Forward primer Stop: 950

Forward primer Tm: 59.70

Forward primer GC%: 40.00

Forward primer Self complementarity: 6.00

Forward primer Self 3' complementarity: 2.00

Reverse primer Sequence (5'->3'): TGGACCAACTTATGGGTGGC

Reverse primer Template strand: Minus

Reverse primer Length: 20

Reverse primer Start: 1500

Reverse primer Stop: 1481

Reverse primer Tm: 59.96

Reverse primer GC%: 55.00

Reverse primer Self complementarity: 5.00

Reverse primer Self 3' complementarity: 2.00

Product length: 575

Primer pair 2

Forward primer Sequence (5'->3'): TGGCATTTGGATCACATATTACCAC

Forward primer Template strand: Plus

Forward primer Length: 25

Forward primer Start: 926

Forward primer Stop: 950

Forward primer Tm: 59.70

Forward primer GC%: 40.00

Forward primer Self complementarity: 6.00

Forward primer Self 3' complementarity: 2.00

Reverse primer Sequence (5'->3'): TGGCTCATGATCTGCACTGG

Reverse primer Template strand: Minus

Reverse primer Length: 20

Reverse primer Start: 1860

Reverse primer Stop: 1841

Reverse primer Tm: 60.11

Reverse primer GC%: 55.00

Reverse primer Self complementarity: 6.00

Reverse primer Self 3' complementarity: 1.00

Product length: 935

Primer pair 3

Forward primer Sequence (5'->3'): TGGCATTTGGATCACATATTACCAC

Forward primer Template strand: Plus

Forward primer Length: 25

Forward primer Start: 926

Forward primer Stop: 950

Forward primer Tm: 59.70

Forward primer GC%: 40.00

Forward primer Self complementarity: 6.00

Forward primer Self 3' complementarity: 2.00

Reverse primer Sequence (5'->3'): GGCTCATGATCTGCACTGGA

Reverse primer Template strand: Minus

Reverse primer Length: 20

Reverse primer Start: 1859

Reverse primer Stop: 1840

Reverse primer Tm: 59.82

Reverse primer GC%: 55.00

Reverse primer Self complementarity: 6.00

Reverse primer Self 3' complementarity: 3.00

Product length: 934

Primer pair 4

Forward primer Sequence (5'->3'): TGGCATTTGGATCACATATTACCAC

Forward primer Template strand: Plus

Forward primer Length: 25

Forward primer Start: 926

Forward primer Stop: 950

Forward primer Tm: 59.70

Forward primer GC%: 40.00

Forward primer Self complementarity: 6.00

Forward primer Self 3' complementarity: 2.00

Reverse primer Sequence (5'->3'): CTGGGTCAGAGAAAGGGACG

Reverse primer Template strand: Minus

Reverse primer Length: 20

Reverse primer Start: 1574

Reverse primer Stop: 1555

Reverse primer Tm: 59.75

Reverse primer GC%: 60.00

Reverse primer Self complementarity: 3.00

Reverse primer Self 3' complementarity: 2.00

Product length: 649

Primer pair 5

Forward primer Sequence (5'->3'): TGGCATTTGGATCACATATTACCAC

Forward primer Template strand: Plus

Forward primer Length: 25

Forward primer Start: 926

Forward primer Stop: 950

Forward primer Tm: 59.70

Forward primer GC%: 40.00

Forward primer Self complementarity: 6.00

Forward primer Self 3' complementarity: 2.00

Reverse primer Sequence (5'->3'): GACCAACTTATGGGTGGCCT

Reverse primer Template strand: Minus

Reverse primer Length: 20

Reverse primer Start: 1498

Reverse primer Stop: 1479

Reverse primer Tm: 59.67

Reverse primer GC%: 55.00

Reverse primer Self complementarity: 4.00

Reverse primer Self 3' complementarity: 2.00

Product length: 573

Primer pair 6

Forward primer Sequence (5'->3'): TGGCATTTGGATCACATATTACCAC

Forward primer Template strand: Plus

Forward primer Length: 25

Forward primer Start: 926

Forward primer Stop: 950

Forward primer Tm: 59.70

Forward primer GC%: 40.00

Forward primer Self complementarity: 6.00

Forward primer Self 3' complementarity: 2.00

Reverse primer Sequence (5'->3'): ACCAACTTATGGGTGGCCTT

Reverse primer Template strand: Minus

Reverse primer Length: 20

Reverse primer Start: 1497

Reverse primer Stop: 1478

Reverse primer Tm: 59.22

Reverse primer GC%: 50.00

Reverse primer Self complementarity: 4.00

Reverse primer Self 3' complementarity: 0.00

Product length: 572

Primer pair 7

Forward primer Sequence (5'->3'): TGGCATTTGGATCACATATTACCAC

Forward primer Template strand: Plus

Forward primer Length: 25

Forward primer Start: 926

Forward primer Stop: 950

Forward primer Tm: 59.70

Forward primer GC%: 40.00

Forward primer Self complementarity: 6.00

Forward primer Self 3' complementarity: 2.00

Reverse primer Sequence (5'->3'): AGCACTTGGAGCATCCTCTT

Reverse primer Template strand: Minus

Reverse primer Length: 20

Reverse primer Start: 1069

Reverse primer Stop: 1050

Reverse primer Tm: 59.01

Reverse primer GC%: 50.00

Reverse primer Self complementarity: 6.00

Reverse primer Self 3' complementarity: 2.00

Product length: 144

Primer pair 8

Forward primer Sequence (5'->3'): TGGCATTTGGATCACATATTACCAC

Forward primer Template strand: Plus

Forward primer Length: 25

Forward primer Start: 926

Forward primer Stop: 950

Forward primer Tm: 59.70

Forward primer GC%: 40.00

Forward primer Self complementarity: 6.00

Forward primer Self 3' complementarity: 2.00

Reverse primer Sequence (5'->3'): ACTTGGAGCATCCTCTTCTGC

Reverse primer Template strand: Minus

Reverse primer Length: 21

Reverse primer Start: 1066

Reverse primer Stop: 1046

Reverse primer Tm: 60.07

Reverse primer GC%: 52.38

Reverse primer Self complementarity: 6.00

Reverse primer Self 3' complementarity: 2.00

Product length: 141

Primer pair 9

Forward primer Sequence (5'->3'): TGGCATTTGGATCACATATTACCAC

Forward primer Template strand: Plus

Forward primer Length: 25

Forward primer Start: 926

Forward primer Stop: 950

Forward primer Tm: 59.70

Forward primer GC%: 40.00

Forward primer Self complementarity: 6.00

Forward primer Self 3' complementarity: 2.00

Reverse primer Sequence (5'->3'): GCACTTGGAGCATCCTCTTCT

Reverse primer Template strand: Minus

Reverse primer Length: 21

Reverse primer Start: 1068

Reverse primer Stop: 1048

Reverse primer Tm: 60.07

Reverse primer GC%: 52.38

Reverse primer Self complementarity: 6.00

Reverse primer Self 3' complementarity: 0.00

Product length: 143

Primer pair 10

Forward primer Sequence (5'->3'): TGGCATTTGGATCACATATTACCAC

Forward primer Template strand: Plus

Forward primer Length: 25

Forward primer Start: 926

Forward primer Stop: 950

Forward primer Tm: 59.70

Forward primer GC%: 40.00

Forward primer Self complementarity: 6.00

Forward primer Self 3' complementarity: 2.00

Reverse primer Sequence (5'->3'): GAGCACTTGGAGCATCCTCTT

Reverse primer Template strand: Minus

Reverse primer Length: 21

Reverse primer Start: 1070

Reverse primer Stop: 1050

Reverse primer Tm: 60.07

Reverse primer GC%: 52.38

Reverse primer Self complementarity: 6.00

Reverse primer Self 3' complementarity: 2.00

Product length: 145

**rs839523 T primer pairs**

Input PCR template: lcl|Query_1

Range: 1 - 9000

Specificity of primers: Primer pairs are specific to input template as no other targets were found in selected database: NCBI Messenger RNA Reference Sequences (Organism limited to Homo sapiens)

Primer pair 1

Forward primer Sequence (5'->3'): AAATTGCACCGCGTGGAAAA

Forward primer Template strand: Plus

Forward primer Length: 20

Forward primer Start: 418

Forward primer Stop: 437

Forward primer Tm: 59.90

Forward primer GC%: 45.00

Forward primer Self complementarity: 6.00

Forward primer Self 3' complementarity: 2.00

Reverse primer Sequence (5'->3'): ACTTATTATTTGCAGATTATTTGCAGCTTA

Reverse primer Template strand: Minus

Reverse primer Length: 30

Reverse primer Start: 979

Reverse primer Stop: 950

Reverse primer Tm: 59.18

Reverse primer GC%: 26.67

Reverse primer Self complementarity: 9.00

Reverse primer Self 3' complementarity: 7.00

Product length: 562

Primer pair 2

Forward primer Sequence (5'->3'): GGGGTGAAACTCAGCAGTGA

Forward primer Template strand: Plus

Forward primer Length: 20

Forward primer Start: 367

Forward primer Stop: 386

Forward primer Tm: 59.89

Forward primer GC%: 55.00

Forward primer Self complementarity: 3.00

Forward primer Self 3' complementarity: 3.00

Reverse primer Sequence (5'->3'): ACTTATTATTTGCAGATTATTTGCAGCTTA

Reverse primer Template strand: Minus

Reverse primer Length: 30

Reverse primer Start: 979

Reverse primer Stop: 950

Reverse primer Tm: 59.18

Reverse primer GC%: 26.67

Reverse primer Self complementarity: 9.00

Reverse primer Self 3' complementarity: 7.00

Product length: 613

Primer pair 3

Forward primer Sequence (5'->3'): AGGGGTGAAACTCAGCAGTG

Forward primer Template strand: Plus

Forward primer Length: 20

Forward primer Start: 366

Forward primer Stop: 385

Forward primer Tm: 59.89

Forward primer GC%: 55.00

Forward primer Self complementarity: 3.00

Forward primer Self 3' complementarity: 3.00

Reverse primer Sequence (5'->3'): ACTTATTATTTGCAGATTATTTGCAGCTTA

Reverse primer Template strand: Minus

Reverse primer Length: 30

Reverse primer Start: 979

Reverse primer Stop: 950

Reverse primer Tm: 59.18

Reverse primer GC%: 26.67

Reverse primer Self complementarity: 9.00

Reverse primer Self 3' complementarity: 7.00

Product length: 614

Primer pair 4

Forward primer Sequence (5'->3'): AGCTTCCATCCCACTCAGGA

Forward primer Template strand: Plus

Forward primer Length: 20

Forward primer Start: 738

Forward primer Stop: 757

Forward primer Tm: 60.25

Forward primer GC%: 55.00

Forward primer Self complementarity: 4.00

Forward primer Self 3' complementarity: 2.00

Reverse primer Sequence (5'->3'): ACTTATTATTTGCAGATTATTTGCAGCTTA

Reverse primer Template strand: Minus

Reverse primer Length: 30

Reverse primer Start: 979

Reverse primer Stop: 950

Reverse primer Tm: 59.18

Reverse primer GC%: 26.67

Reverse primer Self complementarity: 9.00

Reverse primer Self 3' complementarity: 7.00

Product length: 242

Primer pair 5

Forward primer Sequence (5'->3'): AATTGCACCGCGTGGAAAAG

Forward primer Template strand: Plus

Forward primer Length: 20

Forward primer Start: 419

Forward primer Stop: 438

Forward primer Tm: 60.32

Forward primer GC%: 50.00

Forward primer Self complementarity: 6.00

Forward primer Self 3' complementarity: 1.00

Reverse primer Sequence (5'->3'): ACTTATTATTTGCAGATTATTTGCAGCTTA

Reverse primer Template strand: Minus

Reverse primer Length: 30

Reverse primer Start: 979

Reverse primer Stop: 950

Reverse primer Tm: 59.18

Reverse primer GC%: 26.67

Reverse primer Self complementarity: 9.00

Reverse primer Self 3' complementarity: 7.00

Product length: 561

Primer pair 6

Forward primer Sequence (5'->3'): CCTGGCCATCCAGGTTGTTA

Forward primer Template strand: Plus

Forward primer Length: 20

Forward primer Start: 29

Forward primer Stop: 48

Forward primer Tm: 59.67

Forward primer GC%: 55.00

Forward primer Self complementarity: 6.00

Forward primer Self 3' complementarity: 2.00

Reverse primer Sequence (5'->3'): ACTTATTATTTGCAGATTATTTGCAGCTTA

Reverse primer Template strand: Minus

Reverse primer Length: 30

Reverse primer Start: 979

Reverse primer Stop: 950

Reverse primer Tm: 59.18

Reverse primer GC%: 26.67

Reverse primer Self complementarity: 9.00

Reverse primer Self 3' complementarity: 7.00

Product length: 951

Primer pair 7

Forward primer Sequence (5'->3'): AGTGTCTTGGGGTGAAGGGA

Forward primer Template strand: Plus

Forward primer Length: 20

Forward primer Start: 322

Forward primer Stop: 341

Forward primer Tm: 60.40

Forward primer GC%: 55.00

Forward primer Self complementarity: 3.00

Forward primer Self 3' complementarity: 0.00

Reverse primer Sequence (5'->3'): ACTTATTATTTGCAGATTATTTGCAGCTTA

Reverse primer Template strand: Minus

Reverse primer Length: 30

Reverse primer Start: 979

Reverse primer Stop: 950

Reverse primer Tm: 59.18

Reverse primer GC%: 26.67

Reverse primer Self complementarity: 9.00

Reverse primer Self 3' complementarity: 7.00

Product length: 658

Primer pair 8

Forward primer Sequence (5'->3'): ACCGCGTGGAAAAGTCTAGC

Forward primer Template strand: Plus

Forward primer Length: 20

Forward primer Start: 425

Forward primer Stop: 444

Forward primer Tm: 60.67

Forward primer GC%: 55.00

Forward primer Self complementarity: 4.00

Forward primer Self 3' complementarity: 2.00

Reverse primer Sequence (5'->3'): ACTTATTATTTGCAGATTATTTGCAGCTTA

Reverse primer Template strand: Minus

Reverse primer Length: 30

Reverse primer Start: 979

Reverse primer Stop: 950

Reverse primer Tm: 59.18

Reverse primer GC%: 26.67

Reverse primer Self complementarity: 9.00

Reverse primer Self 3' complementarity: 7.00

Product length: 555

Primer pair 9

Forward primer Sequence (5'->3'): AGAGGGGTGAAACTCAGCAG

Forward primer Template strand: Plus

Forward primer Length: 20

Forward primer Start: 364

Forward primer Stop: 383

Forward primer Tm: 59.31

Forward primer GC%: 55.00

Forward primer Self complementarity: 3.00

Forward primer Self 3' complementarity: 2.00

Reverse primer Sequence (5'->3'): ACTTATTATTTGCAGATTATTTGCAGCTTA

Reverse primer Template strand: Minus

Reverse primer Length: 30

Reverse primer Start: 979

Reverse primer Stop: 950

Reverse primer Tm: 59.18

Reverse primer GC%: 26.67

Reverse primer Self complementarity: 9.00

Reverse primer Self 3' complementarity: 7.00

Product length: 616

Primer pair 10

Forward primer Sequence (5'->3'): GTGTCTTGGGGTGAAGGGAT

Forward primer Template strand: Plus

Forward primer Length: 20

Forward primer Start: 323

Forward primer Stop: 342

Forward primer Tm: 59.30

Forward primer GC%: 55.00

Forward primer Self complementarity: 3.00

Forward primer Self 3' complementarity: 2.00

Reverse primer Sequence (5'->3'): ACTTATTATTTGCAGATTATTTGCAGCTTA

Reverse primer Template strand: Minus

Reverse primer Length: 30

Reverse primer Start: 979

Reverse primer Stop: 950

Reverse primer Tm: 59.18

Reverse primer GC%: 26.67

Reverse primer Self complementarity: 9.00

Reverse primer Self 3' complementarity: 7.00

Product length: 657

**rs1816072 C primer pairs**

Input PCR template: lcl|Query_1

Range: 1 - 2471

Specificity of primers: Primer pairs are specific to input template as no other targets were found in selected database: NCBI Messenger RNA Reference Sequences (Organism limited to Homo sapiens)

Primer pair 1

Forward primer Sequence (5'->3'): CATGGGGAATGGTGCTCAGT

Forward primer Template strand: Plus

Forward primer Length: 20

Forward primer Start: 331

Forward primer Stop: 350

Forward primer Tm: 60.03

Forward primer GC%: 55.00

Forward primer Self complementarity: 4.00

Forward primer Self 3' complementarity: 1.00

Reverse primer Sequence (5'->3'): TCAAGATCACACAGATGGAAAGTTG

Reverse primer Template strand: Minus

Reverse primer Length: 25

Reverse primer Start: 974

Reverse primer Stop: 950

Reverse primer Tm: 59.53

Reverse primer GC%: 40.00

Reverse primer Self complementarity: 4.00

Reverse primer Self 3' complementarity: 1.00

Product length: 644

Primer pair 2

Forward primer Sequence (5'->3'): ATGGGCGTGCTCAGAATCAA

Forward primer Template strand: Plus

Forward primer Length: 20

Forward primer Start: 710

Forward primer Stop: 729

Forward primer Tm: 60.04

Forward primer GC%: 50.00

Forward primer Self complementarity: 4.00

Forward primer Self 3' complementarity: 0.00

Reverse primer Sequence (5'->3'): TCAAGATCACACAGATGGAAAGTTG

Reverse primer Template strand: Minus

Reverse primer Length: 25

Reverse primer Start: 974

Reverse primer Stop: 950

Reverse primer Tm: 59.53

Reverse primer GC%: 40.00

Reverse primer Self complementarity: 4.00

Reverse primer Self 3' complementarity: 1.00

Product length: 265

Primer pair 3

Forward primer Sequence (5'->3'): GGATCACTTCGGCTACTGGG

Forward primer Template strand: Plus

Forward primer Length: 20

Forward primer Start: 79

Forward primer Stop: 98

Forward primer Tm: 59.89

Forward primer GC%: 60.00

Forward primer Self complementarity: 4.00

Forward primer Self 3' complementarity: 0.00

Reverse primer Sequence (5'->3'): TCAAGATCACACAGATGGAAAGTTG

Reverse primer Template strand: Minus

Reverse primer Length: 25

Reverse primer Start: 974

Reverse primer Stop: 950

Reverse primer Tm: 59.53

Reverse primer GC%: 40.00

Reverse primer Self complementarity: 4.00

Reverse primer Self 3' complementarity: 1.00

Product length: 896

Primer pair 4

Forward primer Sequence (5'->3'): GGGATCACTTCGGCTACTGG

Forward primer Template strand: Plus

Forward primer Length: 20

Forward primer Start: 78

Forward primer Stop: 97

Forward primer Tm: 59.89

Forward primer GC%: 60.00

Forward primer Self complementarity: 4.00

Forward primer Self 3' complementarity: 1.00

Reverse primer Sequence (5'->3'): TCAAGATCACACAGATGGAAAGTTG

Reverse primer Template strand: Minus

Reverse primer Length: 25

Reverse primer Start: 974

Reverse primer Stop: 950

Reverse primer Tm: 59.53

Reverse primer GC%: 40.00

Reverse primer Self complementarity: 4.00

Reverse primer Self 3' complementarity: 1.00

Product length: 897

Primer pair 5

Forward primer Sequence (5'->3'): CCAAGGTTTTATGGGCGTGC

Forward primer Template strand: Plus

Forward primer Length: 20

Forward primer Start: 700

Forward primer Stop: 719

Forward primer Tm: 60.11

Forward primer GC%: 55.00

Forward primer Self complementarity: 3.00

Forward primer Self 3' complementarity: 2.00

Reverse primer Sequence (5'->3'): TCAAGATCACACAGATGGAAAGTTG

Reverse primer Template strand: Minus

Reverse primer Length: 25

Reverse primer Start: 974

Reverse primer Stop: 950

Reverse primer Tm: 59.53

Reverse primer GC%: 40.00

Reverse primer Self complementarity: 4.00

Reverse primer Self 3' complementarity: 1.00

Product length: 275

Primer pair 6

Forward primer Sequence (5'->3'): GCTGAGTCAAGCACCTGTGA

Forward primer Template strand: Plus

Forward primer Length: 20

Forward primer Start: 156

Forward primer Stop: 175

Forward primer Tm: 60.25

Forward primer GC%: 55.00

Forward primer Self complementarity: 5.00

Forward primer Self 3' complementarity: 3.00

Reverse primer Sequence (5'->3'): TCAAGATCACACAGATGGAAAGTTG

Reverse primer Template strand: Minus

Reverse primer Length: 25

Reverse primer Start: 974

Reverse primer Stop: 950

Reverse primer Tm: 59.53

Reverse primer GC%: 40.00

Reverse primer Self complementarity: 4.00

Reverse primer Self 3' complementarity: 1.00

Product length: 819

Primer pair 7

Forward primer Sequence (5'->3'): TGGGCGTGCTCAGAATCAAA

Forward primer Template strand: Plus

Forward primer Length: 20

Forward primer Start: 711

Forward primer Stop: 730

Forward primer Tm: 60.25

Forward primer GC%: 50.00

Forward primer Self complementarity: 4.00

Forward primer Self 3' complementarity: 1.00

Reverse primer Sequence (5'->3'): TCAAGATCACACAGATGGAAAGTTG

Reverse primer Template strand: Minus

Reverse primer Length: 25

Reverse primer Start: 974

Reverse primer Stop: 950

Reverse primer Tm: 59.53

Reverse primer GC%: 40.00

Reverse primer Self complementarity: 4.00

Reverse primer Self 3' complementarity: 1.00

Product length: 264

Primer pair 8

Forward primer Sequence (5'->3'): TGTCTTGCATGGGGAATGGT

Forward primer Template strand: Plus

Forward primer Length: 20

Forward primer Start: 324

Forward primer Stop: 343

Forward primer Tm: 59.59

Forward primer GC%: 50.00

Forward primer Self complementarity: 4.00

Forward primer Self 3' complementarity: 0.00

Reverse primer Sequence (5'->3'): TCAAGATCACACAGATGGAAAGTTG

Reverse primer Template strand: Minus

Reverse primer Length: 25

Reverse primer Start: 974

Reverse primer Stop: 950

Reverse primer Tm: 59.53

Reverse primer GC%: 40.00

Reverse primer Self complementarity: 4.00

Reverse primer Self 3' complementarity: 1.00

Product length: 651

Primer pair 9

Forward primer Sequence (5'->3'): ACCATAGACCTCCCCTGTGT

Forward primer Template strand: Plus

Forward primer Length: 20

Forward primer Start: 192

Forward primer Stop: 211

Forward primer Tm: 59.58

Forward primer GC%: 55.00

Forward primer Self complementarity: 3.00

Forward primer Self 3' complementarity: 0.00

Reverse primer Sequence (5'->3'): TCAAGATCACACAGATGGAAAGTTG

Reverse primer Template strand: Minus

Reverse primer Length: 25

Reverse primer Start: 974

Reverse primer Stop: 950

Reverse primer Tm: 59.53

Reverse primer GC%: 40.00

Reverse primer Self complementarity: 4.00

Reverse primer Self 3' complementarity: 1.00

Product length: 783

Primer pair 10

Forward primer Sequence (5'->3'): GGTTTTATGGGCGTGCTCAG

Forward primer Template strand: Plus

Forward primer Length: 20

Forward primer Start: 704

Forward primer Stop: 723

Forward primer Tm: 59.55

Forward primer GC%: 55.00

Forward primer Self complementarity: 4.00

Forward primer Self 3' complementarity: 3.00

Reverse primer Sequence (5'->3'): TCAAGATCACACAGATGGAAAGTTG

Reverse primer Template strand: Minus

Reverse primer Length: 25

Reverse primer Start: 974

Reverse primer Stop: 950

Reverse primer Tm: 59.53

Reverse primer GC%: 40.00

Reverse primer Self complementarity: 4.00

Reverse primer Self 3' complementarity: 1.00

Product length: 271

**Rs1816072 T primer pairs**

Input PCR template: lcl|Query_1

Range: 1 - 2471

Specificity of primers: Primer pairs are specific to input template as no other targets were found in selected database: NCBI Messenger RNA Reference Sequences (Organism limited to Homo sapiens)

Primer pair 1

Forward primer Sequence (5'->3'): GATTCTCATTCCAATGGCAACTCTAT

Forward primer Template strand: Plus

Forward primer Length: 26

Forward primer Start: 925

Forward primer Stop: 950

Forward primer Tm: 59.51

Forward primer GC%: 38.46

Forward primer Self complementarity: 6.00

Forward primer Self 3' complementarity: 2.00

Reverse primer Sequence (5'->3'): TTAAGGGCCATGCAGAGAGC

Reverse primer Template strand: Minus

Reverse primer Length: 20

Reverse primer Start: 1885

Reverse primer Stop: 1866

Reverse primer Tm: 60.11

Reverse primer GC%: 55.00

Reverse primer Self complementarity: 4.00

Reverse primer Self 3' complementarity: 2.00

Product length: 961

Primer pair 2

Forward primer Sequence (5'->3'): GATTCTCATTCCAATGGCAACTCTAT

Forward primer Template strand: Plus

Forward primer Length: 26

Forward primer Start: 925

Forward primer Stop: 950

Forward primer Tm: 59.51

Forward primer GC%: 38.46

Forward primer Self complementarity: 6.00

Forward primer Self 3' complementarity: 2.00

Reverse primer Sequence (5'->3'): GGCCATGCAGAGAGCCTAAT

Reverse primer Template strand: Minus

Reverse primer Length: 20

Reverse primer Start: 1880

Reverse primer Stop: 1861

Reverse primer Tm: 59.89

Reverse primer GC%: 55.00

Reverse primer Self complementarity: 4.00

Reverse primer Self 3' complementarity: 2.00

Product length: 956

Primer pair 3

Forward primer Sequence (5'->3'): GATTCTCATTCCAATGGCAACTCTAT

Forward primer Template strand: Plus

Forward primer Length: 26

Forward primer Start: 925

Forward primer Stop: 950

Forward primer Tm: 59.51

Forward primer GC%: 38.46

Forward primer Self complementarity: 6.00

Forward primer Self 3' complementarity: 2.00

Reverse primer Sequence (5'->3'): CACTGTGGTGACAGTGTGGA

Reverse primer Template strand: Minus

Reverse primer Length: 20

Reverse primer Start: 1778

Reverse primer Stop: 1759

Reverse primer Tm: 59.82

Reverse primer GC%: 55.00

Reverse primer Self complementarity: 8.00

Reverse primer Self 3' complementarity: 1.00

Product length: 854

Primer pair 4

Forward primer Sequence (5'->3'): GATTCTCATTCCAATGGCAACTCTAT

Forward primer Template strand: Plus

Forward primer Length: 26

Forward primer Start: 925

Forward primer Stop: 950

Forward primer Tm: 59.51

Forward primer GC%: 38.46

Forward primer Self complementarity: 6.00

Forward primer Self 3' complementarity: 2.00

Reverse primer Sequence (5'->3'): TCACTGTGGTGACAGTGTGG

Reverse primer Template strand: Minus

Reverse primer Length: 20

Reverse primer Start: 1779

Reverse primer Stop: 1760

Reverse primer Tm: 59.82

Reverse primer GC%: 55.00

Reverse primer Self complementarity: 8.00

Reverse primer Self 3' complementarity: 1.00

Product length: 855

Primer pair 5

Forward primer Sequence (5'->3'): GATTCTCATTCCAATGGCAACTCTAT

Forward primer Template strand: Plus

Forward primer Length: 26

Forward primer Start: 925

Forward primer Stop: 950

Forward primer Tm: 59.51

Forward primer GC%: 38.46

Forward primer Self complementarity: 6.00

Forward primer Self 3' complementarity: 2.00

Reverse primer Sequence (5'->3'): AATGTGACACTGGGTCAGGC

Reverse primer Template strand: Minus

Reverse primer Length: 20

Reverse primer Start: 1157

Reverse primer Stop: 1138

Reverse primer Tm: 60.25

Reverse primer GC%: 55.00

Reverse primer Self complementarity: 7.00

Reverse primer Self 3' complementarity: 2.00

Product length: 233

Primer pair 6

Forward primer Sequence (5'->3'): GATTCTCATTCCAATGGCAACTCTAT

Forward primer Template strand: Plus

Forward primer Length: 26

Forward primer Start: 925

Forward primer Stop: 950

Forward primer Tm: 59.51

Forward primer GC%: 38.46

Forward primer Self complementarity: 6.00

Forward primer Self 3' complementarity: 2.00

Reverse primer Sequence (5'->3'): GAGGTTAAGGGCCATGCAGA

Reverse primer Template strand: Minus

Reverse primer Length: 20

Reverse primer Start: 1889

Reverse primer Stop: 1870

Reverse primer Tm: 59.74

Reverse primer GC%: 55.00

Reverse primer Self complementarity: 4.00

Reverse primer Self 3' complementarity: 0.00

Product length: 965

Primer pair 7

Forward primer Sequence (5'->3'): GATTCTCATTCCAATGGCAACTCTAT

Forward primer Template strand: Plus

Forward primer Length: 26

Forward primer Start: 925

Forward primer Stop: 950

Forward primer Tm: 59.51

Forward primer GC%: 38.46

Forward primer Self complementarity: 6.00

Forward primer Self 3' complementarity: 2.00

Reverse primer Sequence (5'->3'): AGGTTAAGGGCCATGCAGAG

Reverse primer Template strand: Minus

Reverse primer Length: 20

Reverse primer Start: 1888

Reverse primer Stop: 1869

Reverse primer Tm: 59.74

Reverse primer GC%: 55.00

Reverse primer Self complementarity: 4.00

Reverse primer Self 3' complementarity: 2.00

Product length: 964

Primer pair 8

Forward primer Sequence (5'->3'): GATTCTCATTCCAATGGCAACTCTAT

Forward primer Template strand: Plus

Forward primer Length: 26

Forward primer Start: 925

Forward primer Stop: 950

Forward primer Tm: 59.51

Forward primer GC%: 38.46

Forward primer Self complementarity: 6.00

Forward primer Self 3' complementarity: 2.00

Reverse primer Sequence (5'->3'): GCAAGGGGGCAATTTGCATT

Reverse primer Template strand: Minus

Reverse primer Length: 20

Reverse primer Start: 1807

Reverse primer Stop: 1788

Reverse primer Tm: 60.32

Reverse primer GC%: 50.00

Reverse primer Self complementarity: 7.00

Reverse primer Self 3' complementarity: 2.00

Product length: 883

Primer pair 9

Forward primer Sequence (5'->3'): GATTCTCATTCCAATGGCAACTCTAT

Forward primer Template strand: Plus

Forward primer Length: 26

Forward primer Start: 925

Forward primer Stop: 950

Forward primer Tm: 59.51

Forward primer GC%: 38.46

Forward primer Self complementarity: 6.00

Forward primer Self 3' complementarity: 2.00

Reverse primer Sequence (5'->3'): TGACACTGGGTCAGGCTGTA

Reverse primer Template strand: Minus

Reverse primer Length: 20

Reverse primer Start: 1153

Reverse primer Stop: 1134

Reverse primer Tm: 60.47

Reverse primer GC%: 55.00

Reverse primer Self complementarity: 7.00

Reverse primer Self 3' complementarity: 3.00

Product length: 229

Primer pair 10

Forward primer Sequence (5'->3'): GATTCTCATTCCAATGGCAACTCTAT

Forward primer Template strand: Plus

Forward primer Length: 26

Forward primer Start: 925

Forward primer Stop: 950

Forward primer Tm: 59.51

Forward primer GC%: 38.46

Forward primer Self complementarity: 6.00

Forward primer Self 3' complementarity: 2.00

Reverse primer Sequence (5'->3'): AGAGAGCCTAATGGGGGAGT

Reverse primer Template strand: Minus

Reverse primer Length: 20

Reverse primer Start: 1872

Reverse primer Stop: 1853

Reverse primer Tm: 59.36

Reverse primer GC%: 55.00

Reverse primer Self complementarity: 3.00

Reverse primer Self 3' complementarity: 1.00

Product length: 948

**rs839523 G/A SNP of the ERBB4**

LOCUS NC_000002 50 bp DNA linear CON 20-MAR-2023

DEFINITION Homo sapiens chromosome 2, GRCh38.p14 Primary Assembly.

ACCESSION NC_000002 REGION: 211951339..211951388

VERSION NC_000002.12 GI:568815596

DBLINK BioProject: PRJNA168

Assembly: GCF_000001405.40

KEYWORDS RefSeq.

SOURCE Homo sapiens (human)

ORGANISM Homo sapiens

Eukaryota; Metazoa; Chordata; Craniata; Vertebrata; Euteleostomi;

Mammalia; Eutheria; Euarchontoglires; Primates; Haplorrhini;

Catarrhini; Hominidae; Homo.

Sequence view

**FASTA SEQUENCE**

TGAGGACTCCAAAAGTTCTAACATTGTACCTGGCCATCCAGGTTGTTATAAAGATATATCTGTCAAAATGGGAAAATATAACATTTTATTTAGTAATCTCTAGTTGGTTTATAATAAAAAATAAAATATGTGAGCCTCTATTCAGACTCTTTCCCCCCAGTCACACAATTCTTAGGAATAAGCATGCTACAAATAATCAATAGAAGAGTAGAATCCTAATGGAGCAAAAGTGGAGAGGAATAGGAAAGGGATATATGGAATTACTCATATCTGGCATCAAATTGGGAACTCTAAGCCACTATGAGTATTCCTAAGACAGTGAGTGTCTTGGGGTGAAGGGATAAATGCCTAGGACAATAGAAAAGAGGGGTGAAACTCAGCAGTGATATGCATTGTTCGTACACAGCAATTTGTTTAAAATTGCACCGCGTGGAAAAGTCTAGCTCTAGGGCTCTGTAATTCCTCCTTCAAACACTGTTCTAATGGTTGAGCTGTTACCTCTTTGAATGCCCTTATCTACCTCTTTGAGCTCTTAAAGAGAGATTATACATACATTCTGACATCATATGGTCTAAAAAGTAAAATTCATTCATTATTAAAAAGTTTTCTTTAAACTTGAATACAATAATTATTTAGTGATTAGATCACAATGATAAATGTGTTGATGGAGGGTGTATTATGACTCTTTTGCCTACAACATATGATAAGATCCATCATTCCAAAACAAACTTATCATTAGCTTCCATCCCACTCAGGAAGTGACTCTTCTTATCAAAAAGTGTAAAATGTCATTGGTTTATCTCAAGATGGAGAAACACATTTATAATGTCAACAGAGAAAACACAGATGAAGCATATTCAGGTAAAAGAAGAAGTTTATTTGTAAAAGAGTAACATTAATAGTAATTTTTCAGTGTCACTTGGCATGGCATTTGGATCACATATTACCA**[C/T]**AAGCTGCAAATAATCTGCAAATAATAAGTGCTCTAGAATGTGTGATAGGTATGAACCAAGAAAAATGCTGAACACATAAAGAAGGACCAACTAATGCAGAAGAGGATGCTCCAAGTGCTCTTATTTACTAGAAAAAGTAAAATATAACTAAGAAAATCTTTGTATTTGCATATTACTAAATATATTTATATTTGAATTAATTGTGTTGTAATATTTACATACTTCTCATTTCAGGGTGTGTATTACATTATTCTCAGACACATGTGTCTTGTCTTCATGGAAGGTCAGATTAAAAGCAATAGCTACTCCCTTGTAAAGATTCTAGAACTGGAGGGATTTGCTGTTATAACTGAGGTTCTAGTGTCCAAAATAAGTCTCTGTCATTATTCATTCTGTTTATTACCCTCTGTTCTTGCAAAATAGTATGCAAAAACTAAGCATTAGCCTAACAAATATCCTTTTCTTATGTGAACTCTGGACATCATTCCATGTTAATGCTGAGAATGCATGTCTAAGCCATTTTGAACAAGGCCACCCATAAGTTGGTCCATAGTAAATTATTTTATGATAATGATAGTGAAGATCCTATTTATCTTAATTGTTACGTCCCTTTCTCTGACCCAGAAAAATAATGTAAATAATAGAAAAAAAATCATAGCAAATCTCTCAACCCAAGATGTGTTGCTCTAATCTTTTATATGTAGAGGGACACATGGAACTGAAGCATTTTAAAAAGAGTTACAGGAGATAAATATAAAATTCTTATATCTAAAAGAAACACATCATGCCTTATTTTGGTCTCTGTATTCTGGTATTCATAGTCACATTCTCTCAGGTATTCCTATAGTCTTTCTGTGAACAAATATCTTGAAACATCTCAAAAGGAAACTCCAGTGCAGATCATGAGCCATGTGTACTGACTACAAATAAAAACAATTACAGGAAACAAGTTAATAATGGTAGGAGTGCTTAATGATGAAAAAGAGTAAATAACGTGGTTTGAGATGATATTGGCAGATCTGGATAAAAAGTGAAGAGATATCTAAAAGCGTAAATTATATGTTATAATGTCTGATAAGGCCACACAACCATGTCAGTTATCCATTGTTCCTCTCCTTTCAGCCATCAGAAAGAATCTCAGTAGCACAACATTTTCCACTGTTTTAGAAAATTTTGAGAGAATAAAAACAACTTTTAAACATATGGACTTATCTATGGGAAAGTATAACGGTAGGAAATGTGGGCTTGTCTGCCAGGCAAAGTTGGACTCCAAACCTAGCCTTTGTGCTTACTAGCTTTGTGGCTTTCATCAGGTTGCTTAACCTCATTTAGCCTCAGTTTCTTCATCTTTAAGAAAGAGATACCAATATTTCTATTATAAAACCGTAATAGGGGTTAGAAGATATAAGAAACCTGTCTGGCAAATAGTAAGTATTTAACAGATGCTAAGTATTACAAATATTATCTCTGATAATAGCCAGGCAGATGAACTGCCTGCAGACAATCTGAGCAGAAAAAAAAAAATTATAATATCATTACATAAGAACTGTTGCTTTTAATCTCTCTGTTATGTAACCTTTGGTACATTTATATCTTGCTTATGCAATTTGCAATTTATTCCTAAATTTCAGAAATGGTTCCTTATACTTTATGTGTGTATATTATGGCCCTAAATATAGACTCTGGCGCTGAACATTTAATAATGGCTGTGGAATGGAATGGAATCGAAAAGTGGAAAGCAATGCTTAAGGTCTTTTTAGATATCGTTTTCCAAGGAAAATAATTTTATGAACTTGCCACTAATACAAAACATTGTTTGAACAAAACAATTATGAATTATGTCCAAGGACCACAGTAGTAAACAGAAATTATAACAATGCCCTAGGGGAGGGATAGCATTAGGAGAAATACCTAATGTAGGTGACGGGTTGATGGGTACAGCAAACCACCATGGCACATGTATACCTATGTAACAAAACTGCACGTTCTGCATATGTACCCCAGAACTTAAAGTATAATTTAAAAAAGTAAATAAAACAAAATAAAATCTAATTTCTGAGTTACTGGCGATGTCTTTGATATTAAATAAAGGTTTTTCTCCAAAAAAAAAAAAAAAAAAAAAAGAAGAAGAAGAATGCCCTAGCAAAGACAAAATCCTGACTCTGGTTCTGGCTCAGTAACAGTTTAGTTCTAGGAAGTCATCCCATTTTGTCAACCAAAGAGTGAATATGCTCCTAAAGATATATCTTCCTCCTCATGGTGTAAGCTTCCGCTCAGTAAAACTGATTGATTTCCATTGTCAATGTAGGTTTCTACAGTTGCTTTGTTCAAGTAGTAAATACAATCGGATATTATTAAAGCCTTGAATGCTTCAAAACTCTTCTAGCCCAAGGGACAATTTGTTTTGCTTATGACATTTGATGATAGAAATCAAAAGATAATGGAAAACAAAAAAATAATTTCCCATACCATATTAGCAATAAGTCCAGGAAAAAAATCATGATTTTTTAAAAATGACAACAAATTTTATAAGCATTTCTCCTGCATAATATTCTAGAGACAATGTGAAATCTGCAGTTATAAACCTGGAATTTTCAATGGATGTCCCTCAATCAAAGATTAGATGGGACGGTATTGGCTCACTGGGTTATCCTTTTACCTCTAGATCATTTCAGAAAAATCAGTCTTCACTATTTTTACATTTATGCTTCATTCCACAACTCTTTTAATAGCTATTAATTTTTCTGTATTTGATATAAATCCCTAATTCTTTTCTTCTTAGTTGACTTTTTAAAAAATTTGATCCTTAATAGAAATGAAGACTAGTTCCTTAAATGTTTCTTCATTTACCTTAAGATTAAAGTGATTACAGTAACACACAATTCCTAAATATTTAATAAGTGTTTAGAATGATTTTATG

CACATTAAAAGCAATTACATGTTCCTAGCTAAGTATGTAAAACTCTTATTCCTAGATTTTTTCCTACTAAAATTATTCATCAAATCCATGTTTATTACTTATTTTTCTTTAAAATTAGCATCTCTTTGAAAATATACCTATCCATTTTTGTTAAAACCTTCTGTAATCAGTCAAAAAGATCCAAATTCTTAACTTCTACTCCAGTGTCTTAAGAACTCATACACAATTAGATATTATCCACAAAAATGCTAATACATTTCATCAGGTAAGAATCAACAAATATAATGCTGATTACTTAAGGTCAATAGACTAAGTCAAATAAACCTGTAGTATCACAGAGAAGAGTAGGGAACAGAGAACTATGGTGGTAAGAGGGGGGGTAATAGAAAGTCCCTTCTGCTGGTAATGACTGCAGAATTTATGTGTTCTCTGACGGTGACAAATGTAGTAGCAAATTGTAGCCTCTCCATCAGTAAAGCTTTCCATTAAATTTCCCTAGGCAATTAATTTCTGTCCCTGCCCTTCACAGCATGTCTGAGAATATCTAAAGCAGAAAACATTCTTAACTATGACATAGCATGAAAAAGTTTACCAGAATAAAAGTACATTAATAACCACAGAGCAAACATTACACATCCCTGCTGCTTGTAGAGATAGTATGTTTGCTTTGGAATTCTGCGTTCATTTTTCCCATTGTTTTTAAAACTCTAAGGATTGTGCCACCCTGGCAAGGAGGGACTCAAAGTCTCATTTAATTAGATTTGAAAAAAAAGTAAGTAATGTGAAATGTCATATACTGACTGGAGTAGAGTGGAATTCATACTACTTACATACTGCGTGAAATAATACTCAAGAGGTAGGAAACCTGGTAAGATCATCCCAACAAACTGCTAACTTTCAGCCATTTCCAGCTAGTCTCTTGGGTGCTAGTGTTGCTATGAGTTCTACTTTCTAAGAATCTGCAGGCCATTTCACTCATCACTGGCCGCTTAGCCAGCTCTCAACCCATGACTTCTTAGCAGTGAACACCTTTAGTTCCATTGAGAAGAGGACTCCTTAGCTCACCTCCCTAGAATTAACCATATTTTTTTTGTCCTTTATATTTTCCTGAGCTGGCTGGAGGGTTAATAGTCCCCCTTCAGATCTCTACATTCCCAATTGATTCTTCATCTCCTGACCTTATCAAGACTGTATTCCTGTATCATAGACTTATTACACTAGAGTTAGGTTTGTAAAATTAAATTAAATTGGCCCAAAGTTGTCTCTGTACCTTGAGTTTCTATACAGCAAACTGTGACCTAATTAGTATATAAACAAACTGCAAACTAACTTAAGAGTATATTCTCATAACAAGTAGCTGAATCTCAGCCAATCATAGCAGCCAGGCCTCAGTCAATCACAGGCTACCAACTGATCACACTATGTCCAAATAAGACAAATGCCACGCTGTAACAATTTAAACTGTTTCTGCATAATACTTACTTGTCCATCTATAAATTCTGCCTGTTCACATTGCTGGGTAGGAATCCCTGAACTACTCCTGATCTGAGTGCTGCCCAATTCATGAACATTCTTTTTTTAAATAAACTCTGCTAAATTTAATTTGTCTAAAGTTTTTCTTTTAACAGATTACACAAACTAAGTTGGAAGCTTTGGTAGATTCATTTACATAATAACACAAACTTTCAATTGTAAGTATACATATTTCAAAAAATTCATAAGTTGAAGATTCTATCATCTCTAAAGTGTGTGTGTGTGTGTGTGTGTGTGTGTGTGTGTATTTGTTTTCTACATATTTATACAGGTAGGCTTATATTTTGAAGCTTGTATTTCAAATAGACATTTTAATAATCTTTAATAACTATTTAAAATATAATACTGAGTTCAATTTTTTTCCTATCAGATAAACACATTAAGCTCCAATGACTTGGGGAATTGACTTTTAACTGAAATTAGATACAACGCATAAATGCAAAAATAGTAAGAATGTTTGGAAACCTTTGAATTAAACTTTGTTTAGATATAGATAGAGCTGAAGTAAAAAGAAATTATTAGTTTTCGGCACTTTCGTACAACTGAAGCTATACCTGAATCAAAGCGTCGTAATGTTTATTAACATTGTCCATATGATGATGAGTATTCTCCACAGAGAGCCAAACTGGTTAGGACGATGCAGCCTAGTGATTAAATTTGGGTTCTGTAGTCTCACAATCTTAGTTTGACTCCTGTCTCCATCACTTATTGACCTTGCGCAAATTATTCAATATTACGTTCCTCTTCTGCACAACAGGAAAAATTCCTGCTAAATGCTCAAAAGTTGAATCAACAAAAGATAATCAATTTCATCATATTATAATTAGTGCAATTATGCCAAAATATTTGTAAATATTTTGTATAAGTTTAGGAATGGTAAACAATATATAAGCAAAAACATTTAATATTAAAATTAATTAAACAATTGGTACTACATAGCTCATAAAGATATAGCTTCAGTCAGATTATCTATTGTTGGCTAAATATGACCACTCCCTAAAATAAATTTATTCATGTATATATTTGTTTGTTTATGAGACAGATTCTTGCTCTGTTGCCCAGGCTGGAGCGCAGTGGTGTGATCACAGCTCACTGCAGTCTTGAACTCTGGGTTCAAGAGATCCTCCCACCTCAGATTCCTGTGTCTAGGACTACAGCTACACAACACCACACCAACCTAATTTTTTTTCCAGTAGAGATGGGGGTCTCATTATGTTGCTGTGGCTGGTTTTGAACTCCTCGCCTCAAGCAATACTCCTGCCTTTGCCTCCCAAAGTGCTAGGATGACAGATGTGAGCCACTGTGCCCAGCCAAACCCCCATAATGTATACGCTGAAGTCCTAACCCATACTACCTCAGAATGGGACTGTATTTGGAGATAGGGTTTTTAAAGAGGTAATTAGGCTAAATTGAGGTTATTAAGTCGGGCCCTAATCCAATATAACCAGTGTTTTTCAAAGAAGAAAAGATGAGGGCACAGACATGACAAGAAAAAAGACCATGAGAAAACACAGAGAGAAGTTGGTCATCTACAAGCCAAGGAGAGAGATCTCAGAAGAAACCAACGCTACAGAGCCCTTGATCTCATACTTCCAGCTCCAGAATTGTGAGAAAATAAATATCTGTTGTTTAAGCCACCCAGTCTGTGGTACTTTGTTATGGCATTCCTAGCAAACTAAAACACTGCCTAAGTTCACATCCTAGCTTTTAGCAGTATTATGTTGGGTACATTATTTAACCTTTCTGTGCCTCAGGATCTTTATCTGTAAAATGGGGATGTTAAGGTCTCCAAATCCTAGGATTATTTTGAGAAGTGTATCTCTATGACTTGCTTAACAGAAGGTCTGGCTCATAGCTATCACTCAGTATTAGTTTCTATTGTTTTTCTGAATCCAGAACTCCTTTTCAATATGTTCCCTGTGTTGCACAGCATATATAAAACTGTATTTTTCTATTTCTAAAGGAATAACTACACATCACCAAAGTTTAGATGAGAAAACTGCTTGCTAGCATTTCAATGTACAATTAAGAGTGACAGTTTCATGTCTGAGGTCTTTTATAAATGTGATGTTTTCCCATTCAAAATTTAACGAAAGAAAATGGGCCTCTATGAAATTATGGCTGACAACAGCTGCCTCTGTTGAAAAGCATATGGGAAATACCCTTTGAGGATGCAAAAGTTAGAGGTGACTGATTCTGTGACTAGTCTGTTGTTATTTTTAAATGAAAAGCATGAGCAATCTCAGAAATAAGAGAGCCAAAGTATCAGGCCTGGATTTGACAAGCCTGCCAAGACACACTTCAATATCTGTCTAAATATTTGGCCATAAAAAACAC

AAAGCACAGCCTTAATTAATTTATAAAAAAACAAATATTTGCTTTAGTCATCTCCTCTAGTGACAACAAGGTCTGATGTTACTTATGATGCTCTTAATATTAAATTCAAACATACGCAATAGGCATTCTATGGACATCTGTGGATACAATCAGGACTACAAATATAACAAAAATTGAGGCCATGGACTTGTAGACAAGAAATTTAAATGTTTTTCTTATTTAAGTATCCTTATGCAAGTCACAACTTTCTAAGTAGCTATTTACTATTTCAAATAGTACAAATTTTCAAATAATTACATTAACTTCTATTAATTAGTGTTTTCTGTGTGTGAGGCATTCTTATGTCTTCTGTCACCTACTGGTGAAATCACTCTGACTATTACCTCTGTTTTATAAATGAAGTAACTGCAGTGAAATAACTTGCCCAAAGTCATATAGACAAAAAGTGATGGAGGAAATATTAAAACAACAACAAGATCGCTCTTATTCCCAAGATCAAAATCCCAATCCCTTTGAAGTATTTTTTCAAAAAGTGGTACCAATTACTTCCTTGGTTAAAGTACTATCCACGGCAAAATGGTCTAAAATTTTGATTTAAGAATAACATTCTCTTTCTAAGGTTTATTTATGAGAAAAATATTAGCTAAGAAAGAGTAATTTCTCAAAAGTAACTGCAGCATGCTTCAGATATTTATAACTGCAAAATCTGCTTTAAAAGGTACTTTAAGTATCCTAAAAGTCTCAAAATCAGCATTCTCTTAGGTGCCTTCTCTTAGGTGCCTTCTCTTAGATGCTATCTGACCTTTTCAAACATACATAGAATCAAATGAGATTCTGTTCATAAATTAATAATGCAAATATAACCAAACAATCCTAACGTAAATGTATTTTTAAATTTGCACTTGACTTTTCTTCCTCAAAAAAGTCTTTAAGTCTCTATGCTTTCACCCTCATTCATCCATCCATCCATCCACCCACCCATACATCTATTCATCCTTCCAGCCAATTATCTATTCTACAAATGTTTATTGTCCCCCTCTATTTGAGAAACATTGTTCAAGTTGCTGGAGACACAGTGGTGAGCAAAATAGACATGGTTCTTAATCTTACGGTGGTTACTTCCTTTATCATCTCCAATCTTCGTCTAATGACAAGATTGCCACTAAGAAAATGATGGCTTGCCAAGAAAGATTTACAATAACTTGCAAGTGAATGCCTGTATCTTTGTTTCTTTAAGAGTAAACCATTTGTTTTAAAATT

**rs1816072-GABRB2**

>ref|NG_047050.1|:220476-220476 Homo sapiens gamma-aminobutyric acid type A receptor subunit beta2 (GABRB2), RefSeqGene on chromosome 5 [marker=NG_047050.1:g.220476T_C]

Sequence view

**FASTA SEQUENCE**

AAAGGCTTTGCTTTTATTTTGAATGAAATAGCCATTGACTGAGCATAAAAGTGGCATGATTTGATTTCTGTTTTAAAGGGATCACTTCGGCTACTGGGTTAAAATTAAATTATGTGGCTCAAGGATGGGAGGAAGGAGAAGAAATGACCTCTCAGGCTGAGTCAAGCACCTGTGATATATACACTTGCAGTACCATAGACCTCCCCTGTGTGTAGACTAACCACAGTTATAACTTTTGTTTTCTTAGTGGTGTGATGTCCTTTTCCCCTTCAGCAATGAAAGATCCATGGAGGAAAGGTCCATATCTAGTTTTATATCTGCAGTGTCTTGCATGGGGAATGGTGCTCAGTAAACATTTGCTGAATGAAAGAAGGAATAAAATTTGCAAATATCCCATTTTTCCAAAGTTGAAACATGTCTTTTTTGTATCGATTACAGGAACAATAAATAATTCATTAACTTTTTAGAAGACATTTTTAAAATGTCTTTATGTGAGCAATTTATTACTAGTTTCAGAAATACTTTAAAATGAGATTTAGATAAGTAGTAAAATCAGCAATGTCAGTTTTATTATATACGTATGATACAGGATCATCCGTAGTTGAAATGATGAAGAGAAAATTGAAACTGGAAAATGTCTCAAAATGACAGCTATAAAGAGGACACGAGAAATGTAAGATACCTGTATCTAAAGCAAGACCAAGGTTTTATGGGCGTGCTCAGAATCAAATGCTCAGATTTATTTTTTTATTCTGTAGCTGTTTCTTGGTGGAGTATAAAAACAAAATAGAAACTATGACATTATTACTACTGAGATAAAATTAAATCAGAAAACTGTGTCCCCCCAAAATCATAGGAATGAAATTTAACCTTCAGGATGCGTTATTGGATAGTGTTTAAGGGCCTGGATTCCTGAGTCAGAAAGATTCTCATTCCAATGGCAACTCTA**T**AACTTTCCATCTGTGTGATCTTGACCTCTGAGCTTTGTCTATTTTCACATCACTATTTTGCTACAAAGCTGGACTTTATCATATTCAGGACCTTGGACTTTCAGTGCATTTACCTTCCGATTACAGCTTAGAGGGAAACTACTTCCCACTGTCAAGAGATACCAACTCAACAGGACAAACAATTACAGCCTGACCCAGTGTCACATTTTCCATAAGCAGTCCTAGAATGCATCTGTGTTATTTATCTAGGCATTATATGCCTAATTGTCCGATCCATATACATGAGGGCTATGATTCCTGCTTCTCTGTTACCCTCCACTCCAAATTCCCAGATGTTTATAATCATTTATTGATATTGCCTGTATAATGTATTCTTTAATTTCAGTACCCTTGCTGTTATTTTAATTGACTTCACTTCCATCATTCAGTTGGATTACTCTGAAAACCTCTTAACTGGCATTCTTTTTTTTTTTTTTTTTTTTTGAGACGGAGTCTTGCTGTGTGGCCCAGGCGGGAGTGCAGTGGCGCAATCTCGGCTCACTGCAAGCTCCGCCTCCCGGGTTCACGCCATTCTCCTGCCTCAGCCTCCCGAGTAGCTGGGACTACAGGCGCCCGCCATCACGCCCGGCTAATTTTTTTGTATTTTTAGTAGAGACAGGGTTTCACCGTGTTAGCCAGGATGGTCTCGATCTCCTGACCTCGTGATCCACCCGCCTCGGCCTCCCAAAGTGCTGGGATTACAAGCGTGAGCCACCGCGCCCGGCCAACTGGCATTCTTTACTCCTGCCATGCCTGCTTCTCCCCATCATCCACACTGTCACCACAGTGATTGTTCTAAATGCAAATTGCCCCCTTGCTTGAAGCTGGCCCCTGCTCCACATTGCCTTTAATTTTAAGTTCAAACTCCCCCATTAGGCTCTCTGCATGGCCCTTAACCTCCAGTCTTCTCACGATTCTTCCCATCCTGTCTTGCAATGTACCCACAATGATTTTTCCGAGATCATGGTAATGGACTGTGTACTCTCCCCCAAATCCAACCTTGATATAAAAGCTCTCTCCACCAGCAGTCCCCATAAGTCAAAAATGACTTTTGACCCCCAGTTCTGGGTTGCCTGGGTGACTTTCACTCATTTTTCAGAAATCAGCTCAAAAGTCTCTATTTCCTAACCTCATCCCTGGCACATCAGAAAGTTGACCTAGAGTCCTCTCCATATTCTGAACTCTTACCTTACACATGTGCCCATCATAGCATTTAATTCACGTTTTAACCCCGCACTAGATATATAACTATAAATACAAAAATTTAATTAAAAGAAGGGATCAAACGTTCAGTGATCACTGTAAGCTAATTTATACCTATATCCATTCCCTATCCTAGGGCTGGCATGCAATAGTAAAGGCCCAATAAATAGATTGTGGCATATATGAATGAGAAAATGAATATAGATCACCTTTCCAGCTATTAAGAAAGAAGAAAAGAGATAGGAACATTATTACTCTAACTCTCCCATTTTTTTTCAG

**Supplementary file 2**

| **Table S1: List of Primers Used in the Study with Reference Numbers and Suppliers** | | |
| --- | --- | --- |
| **Primers Name** | **Reference Number** | **Company Name** |
| rs839523CF-Jinan1 | 8821476539-000210 | Ibrahadad Et Fils |
| rs839523CR-Jinan1 | 8821476539-000220 | Ibrahadad Et Fils |
| rs839523TF-Jinan1 | 8821476539-000230 | Ibrahadad Et Fils |
| rs839523TR-Jinan1 | 8821476539-000240 | Ibrahadad Et Fils |
| rs1816072TF-Jinan1 | 8821476539-000250 | Ibrahadad Et Fils |
| rs1816072TR-Jinan1 | 8821476539-000260 | Ibrahadad Et Fils |
| rs1816072CF-Jinan1 | 8821476539-000270 | Ibrahadad Et Fils |
| rs1816072CR-Jinan1 | 8821476539-000280 | Ibrahadad Et Fils |

| **Table S2: Logistic Regression Analysis of Participant Status (Schizophrenia vs. Healthy Control) with Allele Variables as Independent Variables** | | | |
| --- | --- | --- | --- |
| **Factor** | **OR** | **95% CI** | **P value** |
| **ERBB4 rs839523** |  |  |  |
| Allele C | 6.724 | 0.743; 60.862 | 0.090 |
| Allele T | 0.848 | 0.366, 1.965 | 0.701 |
| **GABRB2 rs1816072** |  |  |  |
| Allele T | 0.001 | 0.0001; 1.000 | 1.000 |
| **Gender** | 1.489 | 0.649; 3.416 | 0.347 |
| **Age** | 1.019 | 0.979; 1.060 | 0.366 |
